# The Impact of Glucomannan, Inulin, and Psyllium Supplementation (Soloways^TM^) on Weight Loss in Adults with FTO, LEP, LEPR, and MC4R Polymorphisms: A Randomized, Double-Blind, Placebo-Controlled Trial

**DOI:** 10.3390/nu16040557

**Published:** 2024-02-17

**Authors:** Evgeny Pokushalov, Andrey Ponomarenko, Claire Garcia, Inessa Pak, Evgenya Shrainer, Mariya Seryakova, Michael Johnson, Richard Miller

**Affiliations:** 1 Center for New Medical Technologies, Novosibirsk 630090, Russia; ponomarenko_av@cnmt.ru (A.P.); inesspak@yandex.ru (I.P.); shrayner_ev@cnmt.ru (E.S.); serma_v@mail.ru (M.S.); 2 Scientific Research Laboratory, Triangel Scientific, San Francisco, CA 94101, USA; info@triangelcompany.com (C.G.);

**Keywords:** obesity, genetic polymorphisms, dietary fibers, glucomannan, inulin, psyllium, weight management, personalized nutrition

## Abstract

This study aimed to determine the impact of a fiber supplement on body weight and composition in individuals with obesity with specific genetic polymorphisms. It involved 112 adults with obesity, each with at least one minor allele in the FTO, LEP, LEPR, or MC4R polymorphism. Participants were randomized to receive either a fiber supplement (glucomannan, inulin, and psyllium) or a placebo for 180 days. The experimental group showed significant reductions in body weight (treatment difference: −4.9%; 95% CI: −6.9% to −2.9%; *p* < 0.01) and BMI (treatment difference: −1.4 kg/m^2^; 95% CI: −1.7 to −1.2; *p* < 0.01) compared to placebo. Further significant decreases in fat mass (treatment difference: −13.0%; 95% CI: −14.4 to −11.7; *p* < 0.01) and visceral fat rating (treatment difference: −1.3; 95% CI: −1.6 to −1.0; *p* < 0.01) were noted. Homozygous minor allele carriers experienced greater decreases in body weight (treatment difference: −3.2%; 95% CI: −4.9% to −1.6%; *p* < 0.01) and BMI (treatment difference: −1.2 kg/m^2^; 95% CI: −2.0 to −0.4; *p* < 0.01) compared to heterozygous allele carriers. These carriers also had a more significant reduction in fat mass (treatment difference: −9.8%; 95% CI: −10.6 to −9.1; *p* < 0.01) and visceral fat rating (treatment difference: −0.9; 95% CI: −1.3 to −0.5; *p* < 0.01). A high incidence of gastrointestinal events was reported in the experimental group (74.6%), unlike the placebo group, which reported no side effects. Dietary supplementation with glucomannan, inulin, and psyllium effectively promotes weight loss and improves body composition in individuals with obesity, particularly those with specific genetic polymorphisms.

## 1. Introduction

Obesity, a global epidemic, continues to escalate in prevalence, significantly contributing to various metabolic disorders such as type 2 diabetes, hypertension, dyslipidemia, and cardiovascular diseases, thereby reducing overall wellbeing and life expectancy [1,2,3,4,5]. The complexity of obesity underlines the need for novel and diverse strategies to curb its growth. Currently, management involves caloric restriction and aerobic exercise, targeting fat reduction and increased energy expenditure [6]. However, the effectiveness of diets and exercise is often limited by factors like genetic predisposition, adherence issues, and weight regain post-dieting [7,8,9].

Pharmacotherapy has emerged as an adjunct or alternative when lifestyle modifications are insufficient [10], but it is not devoid of safety concerns and side effects [11]. Consequently, natural dietary supplements, particularly high-fiber options, have gained popularity. Viscous fiber, for instance, has demonstrated significant impacts on body weight, BMI, and waist circumference, independent of calorie-restricted diets [12]. High-fiber diets are also linked to reduced risks of certain cancers, heart disease, diabetes, stroke, and overall mortality [13,14]. Dietary fibers like glucomannan, inulin, and psyllium, through mechanisms such as appetite suppression and satiety enhancement, contribute variably to weight loss and beneficial metabolic changes [15,16,17,18,19,20,21,22,23,24,25,26,27,28].

Glucomannan, inulin, and psyllium are key nondigestible carbohydrates (NDCs) in our study. Glucomannan, derived from the Amorphophallus konjac tuber, is known for its significant water absorption ability, aiding weight loss by delaying gastric emptying and enhancing satiety [18,19]. Inulin, a soluble and fermentable fiber, has shown potential in promoting weight loss and reducing triacylglycerol (TAG) levels in individuals with obesity and dyslipidemia [20,21,22]. Psyllium, a viscous, gel-forming fiber, is noted for its lesser fermentation, thereby reducing gastrointestinal discomfort. It can impact body composition through varied mechanisms, including modifying gastric emptying, satiety enhancement, influencing intestinal hormone secretion, and altering glycemic response [24,25,26,27,28].

Moreover, the genetic aspect of obesity, particularly the role of specific polymorphisms in genes like obesity-associated (FTO), leptin (LEP), leptin receptor (LEPR), and melanocortin 4 receptor (MC4R) genes, has been extensively studied. These polymorphisms are associated with obesity-related traits such as BMI, hip circumference, and cardiometabolic traits [29,30,31,32,33,34,35,36]. Understanding these genetic factors is vital for personalizing diet and exercise regimens and enhancing obesity prevention and treatment strategies.

Our study, a placebo-controlled randomized trial, delves into the efficacy of specific dietary fiber supplements—glucomannan, inulin, and psyllium—in individuals with obesity-related genetic polymorphisms within genes such as FTO, LEP, LEPR, and MC4R. These supplements are known to influence weight management through mechanisms like appetite suppression and enhanced satiety [37]. Simultaneously, the selected genetic polymorphisms are implicated in obesity predominantly due to their influence on eating behaviors, specifically predisposition to overeating [38]. By scientifically correlating these genetic markers with the physiological effects of dietary fibers, our research aims to establish a more nuanced understanding of obesity management, tailored to individual genetic profiles.

## 2. Materials and Methods

We conducted a randomized, double-blind, parallel-group clinical trial in which a single supplement regimen, containing a combined formulation of glucomannan, inulin, and psyllium fibers, was compared to a placebo. The study protocol was approved by the local ethics committee and conducted in accordance with standard institutional operating procedures and the Declaration of Helsinki. All participants enrolled in the study provided written informed consent. The study was registered with ClinicalTrials.gov (NCT06188832).

### 2.1. Patient Population and Design

Patients were eligible based on the following criteria:

Inclusion criteria:

- Healthy participants aged between 40 and 60 years;
- BMI of 25 or greater and no more than a 3% change in body mass within the last three months;
- Presence of at least one minor allele in any of the following genetic polymorphisms: FTO (rs9939609; T > A), LEP (rs2167270; G > A), LEPR (rs1137101; A > G; Gln223Arg), and MC4R (rs17782313; T > C) [38].

Exclusion criteria:

- Individuals who have taken any prescribed medications or dietary supplements in the two weeks prior to the study;
- Those with a clinically significant history of major digestive, liver, kidney, cardiovascular, hematological diseases, diabetes, gastrointestinal disorders, or any other serious acute or chronic medical conditions.

**Figure 1:**
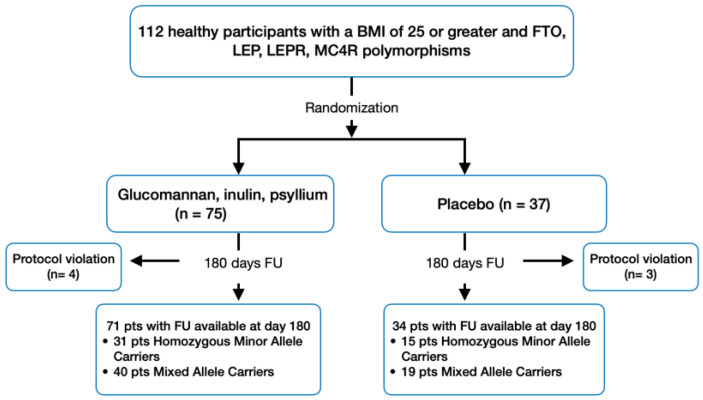
Study design and patient flow.

In the study, participants with polymorphisms in the FTO, LEP, LEPR, and MC4R genes were identified from the database of the Center for New Medical Technologies’ genetic laboratory. Following their consent to participate and if they met the inclusion and exclusion criteria, these participants were enrolled in the research. A total of 112 participants were enrolled in the study. Participants were randomly assigned in a 2:1 ratio, through the use of an interactive web-based response system, to receive glucomannan, inulin, and psyllium (*n* = 75) or matching placebo (*n* = 37), in addition to lifestyle intervention (Figure 1). Blinding was maintained for both researchers and participants. Participants were instructed to consume three powder bags daily, one 30 min before each main meal (breakfast, lunch, dinner), by dissolving the content in a glass of water to ensure adequate hydration. The powder bags, visually identical to maintain blinding, were sourced and provided by S.Lab (Soloways), LLC (Novosibirsk, Russia). Each active powder bag contained 1 g of glucomannan (with an average molecular weight of approximately 200,000 Da, as high molecular weight is associated with increased satiety [39], 1 g of inulin (predominantly composed of long-chain inulins with an average degree of polymerization of 10 for optimal prebiotic effects [40]), and 3 g of psyllium (with 70% soluble fiber [41]), with no additional fillers, totaling 5 g of active ingredients. The placebo powder bags were specifically engineered to not only mimic the active powder in terms of texture and volume, using maltodextrin and rice flour, but also to match the taste and energy content closely. Each bag weighed 5 g, identical to the active bags. This careful formulation ensured comparability in physical characteristics, taste, and energy value, facilitating an accurate assessment of satiety responses and appetite regulation between the active and placebo interventions. The study duration was 180 days. To monitor adherence to the supplementation protocol, a researcher conducted weekly check-ins. Participants were required to return all powder bags, used and unused, to enable precise compliance assessment with the supplementation regimen.

All participants received individual counseling sessions every 4 weeks to help them adhere to a reduced-calorie diet (500 kcal deficit per day relative to the energy expenditure estimated at the time they underwent randomization) and increased physical activity (with 150 min per week of physical activity, such as walking, encouraged). Both diet and activity were recorded daily in a diary or by use of a smartphone application or other tools and were reviewed during counseling sessions.

In this study, S.Lab (Soloways), a pharmaceutical company, contributed solely by manufacturing the dietary fiber supplements (glucomannan, inulin, and psyllium) used in the research. S.Lab (Soloways) did not participate in the design, execution, or financing of the experiment, beyond providing the required supplements. The entire study was independently conducted by a research team from the Center for New Medical Technologies and the Scientific Research Laboratory at Triangel Scientific. This arrangement ensured that the study’s outcomes were not influenced by commercial interests, maintaining the integrity and independence of our research.

### 2.2. Study Endpoints and Assessments

The coprimary end points were the percentage change in body weight from baseline to day 180 and achievement of a reduction in body weight of 5% or more from baseline to day 180. Secondary endpoints included BMI and body composition, which was assessed using bioelectrical impedance analysis (BIA). Measurements were performed with an InBody 770 device from InBody Co., Ltd. (Seoul, Republic of Korea), utilizing multiple frequencies for increased accuracy and detailed body composition analysis, specifically measuring fat mass, fat-free mass, and visceral fat rating. The device was calibrated before each measurement session according to the manufacturer’s instructions. Participants were advised to maintain standard hydration status and to avoid eating or exercising for at least 3 h before the measurement to ensure the consistency and reliability of the results.

Safety was meticulously evaluated by systematically recording adverse events during the treatment period, up to day 180. Adverse events were identified through a structured questionnaire administered at regular intervals (weekly) throughout the study. The questionnaire was specifically designed to capture information on a wide range of potential adverse effects, including but not limited to gastrointestinal discomfort, allergic reactions, and other systemic symptoms. Participants were asked to report the occurrence, duration, and severity of any symptoms they experienced. The severity of adverse events was scored using a standardized scale, ranging from mild to severe, to ensure consistent and objective evaluation.

Concurrently, the effect of the fiber supplement regimen comprising glucomannan, inulin, and psyllium, compared with the placebo, on appetite was assessed using mean postprandial participant-reported visual analogue scale (VAS) appetite ratings following a standardized breakfast meal at day 180. These scores reflect the absolute change from pre-breakfast (fasting) levels, where negative values indicate a decrease in hunger or prospective food consumption, and positive values signify an increase in fullness or satiety. The breakfast was nutritionally balanced, comprising 30 g of protein (from sources such as eggs, low-fat cottage cheese, or chicken breast), 50 g of carbohydrates (wholegrain bread or oatmeal), and 10 g of fats (avocado or olive oil), totaling approximately 350 calories. The supplement was administered 30 min before the breakfast meal, allowing for a comprehensive evaluation of hunger, fullness, satiety, prospective food consumption, and overall appetite suppression score.

In the study, participants were categorized into two distinct genetic groups for our predefined sub-analysis: the homozygous minor allele carriers subgroup includes participants who have two copies of the minor allele for at least one of the specific genes under study; the mixed allele carriers subgroup comprises all other participants. This category is not limited to heterozygous genotypes for all genes but includes any genetic configuration that is not homozygous minor. These categorizations, as pre-specified in the study protocol, were instrumental in facilitating a detailed assessment of the therapy’s effectiveness in relation to these specific genetic profiles.

### 2.3. Sample Size Calculation and Statistical Power

For our randomized, placebo-controlled trial, the primary endpoint was to achieve a reduction in body weight of 5% or more from baseline to day 180. We had anticipated a weight reduction of 7% in the active-substance group and 3% in the placebo group, with the proportion of participants achieving this reduction being 58% and 11%, respectively. The anticipated data for the calculation were derived from a previously conducted extensive study [37]. The trial was designed with a 2:1 randomization ratio (active substance to placebo).

The sample size calculation, which incorporated a two-sided significance level of 5% and aimed for a power of 90%, was adjusted to account for the frequency of FTO, LEP, LEPR, and MC4R polymorphisms in heterozygotes and homozygotes for the minor allele (according to Hardy–Weinberg equilibrium). To account for a potential dropout rate of 20%, these numbers were further adjusted, rounded up to the nearest whole number. Consequently, we determined that enrolling 75 participants in the active-substance group and 37 in the placebo group was necessary to maintain the desired power of the study. This adjustment resulted in a total sample size of 112 participants for the trial.

### 2.4. Statistical Analyses

The statistical analyses for this clinical trial were designed to assess the efficacy and safety of glucomannan, inulin, and psyllium supplementation compared to placebo in altering body weight, body composition, appetite ratings, and other health parameters. Our analysis adhered to the standards of high-quality medical journals and was performed using the latest statistical software.

The primary efficacy analyses were based on the intention-to-treat population, which included all participants who were randomized. The percentage change in body weight from baseline to day 180 and the proportion of participants achieving a reduction in body weight of 5% or more from baseline to day 180 were the co-primary endpoints. These were analyzed using an analysis of covariance (ANCOVA) model, with the baseline value as the covariate and treatment group as the factor.

Secondary endpoints, including changes in BMI, body composition, and appetite ratings, were analyzed similarly using the ANCOVA model. A two-sided significance level of 0.05 was used for all analyses.

Participants were categorized into two genetic groups for a predefined sub-analysis: homozygous minor allele carriers and mixed allele carriers. The efficacy of the intervention within these subgroups was analyzed using the same ANCOVA model, with the addition of the subgroup as a factor.

Safety data were summarized descriptively, and the incidence of adverse events was compared between treatment groups using the Chi-square test or Fisher’s exact test, as appropriate. Bonferroni correction was applied for multiple comparisons to control the family-wise error rate. Missing data were handled using multiple imputation techniques to reduce bias and increase the robustness of our results. Sensitivity analyses were conducted to assess the impact of missing data on the study conclusions.

Sample size was calculated based on anticipated differences in the primary endpoint, with adjustments for dropouts. The power of the study was set at 90%, with a two-sided significance level of 5%. This ensured adequate power to detect clinically meaningful differences between the treatment and placebo groups.

All analyses were performed using statistical software SAS version 9.4 (SAS Institute), which is widely recognized for its reliability and accuracy in the scientific community.

Our statistical analysis adhered to the rigorous standards required for high-quality clinical research, ensuring that our findings are reliable and can be confidently applied to the broader population.

## 3. Results

In this study, 112 individuals were enrolled and randomized to receive either a combination of glucomannan, inulin, and psyllium (*n* = 75) or a placebo (*n* = 37). A subgroup analysis in the experimental group was also conducted on participants identified as homozygous minor allele carriers (*n* = 33) or mixed allele carriers (*n* = 42). The completion and adherence rate for the trial was high, with 93.7% of participants finishing the study and adhering to the treatment protocol (Figure 1).

Demographic and baseline clinical characteristics were well balanced across the treatment and placebo groups (Table 1). The majority of participants were female, accounting for 71.6% of the study population, with a pooled mean age of approximately 46 years. The average body weight was 89.9 kg, and the mean BMI was 30.9 kg/m^2^, indicating an overweight status among the participants.

The subgroup analysis aimed to investigate the influence of genetic variation on baseline characteristics. Homozygous minor allele carriers had a slightly higher, but not statistically significant, mean body weight (91.4 ± 11.1 kg) and BMI (31.1 ± 3.8 kg/m^2^) compared to mixed allele carriers (87.8 ± 9.6 kg and 30.5 ± 2.9 kg/m^2^, respectively). Furthermore, there were no significant differences in the lipid profiles, including total cholesterol, LDL-C, HDL-C, and triglycerides, between the genetic subgroups at baseline.

### 3.1. Change in Body Weight

**Figure 2:**
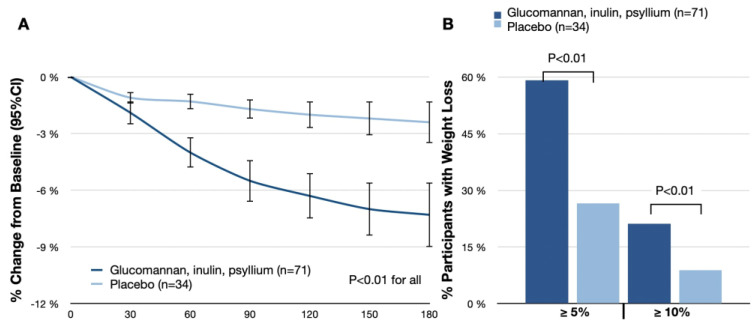
(**A**) Mean percentage change from baseline in body weight; (**B**) percentages of participants who had body weight reductions of at least 5% and 10% from baseline to day 180.

In the experimental group, weight loss was observed from the first postrandomization assessment (day 30) onward, reaching the lowest point at day 180 (Figure 2). The change in body weight from baseline to day 180 was −7.3% (95% CI: −9.0–5.6%) with glucomannan, inulin, and psyllium, compared with −2.4% (95% CI: −3.5%−1.3%) with placebo (treatment difference, −4.9%; 95% CI: −6.9–2.9%; *p* < 0.01; Figure 2A).

Among the participants at the day-180 visit, the thresholds of losing 5% or more and 10% or more of baseline body weight were achieved by 59.2% (42 participants) and 21.1% (15 participants) in the experimental group, respectively, compared with 26.5% (9 participants) and 8.8% (3 participants) in the placebo group (*p* < 0.01 for both thresholds; Table 2; Figure 2B). The absolute change in body weight from baseline to day 180 was −6.5 kg (95% CI: −7.2 kg to −5.9 kg) in the experimental group as compared with −2.2 kg (95% CI: −2.4 kg to −2.0 kg) in the placebo group (treatment difference, −4.3 kg; 95% CI: −5.2 kg to −3.5 kg; *p* < 0.01).

**Figure 3:**
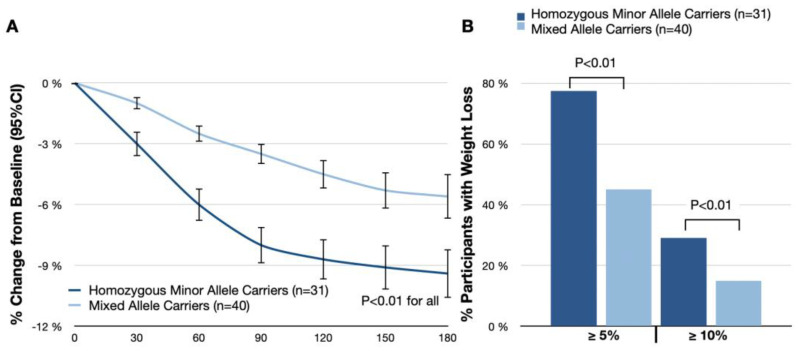
(**A**) Mean percentage change from baseline in body weight; (**B**) percentages of participants who had body weight reductions of at least 5% and 10% from baseline to day 180.

In the subgroup analysis, homozygous minor allele carriers exhibited a more significant reduction in body weight from baseline to day 180 of −9.4% (95% CI: −10.6–8.2%), as opposed to a −5.6% (95% CI: −6.7–4.4%) reduction in mixed allele carriers (treatment difference, −3.2%; 95% CI: −4.9–1.6%; *p* < 0.01; Figure 3A). Furthermore, 77.4% (24 participants) in the homozygous subgroup and 45.0% (18 participants) in the mixed allele carriers group met the threshold of losing 5% or more of baseline body weight. Additionally, 29.0% (nine participants) in the homozygous subgroup and 15.0% (six participants) in the mixed allele carriers group met the threshold of losing 10% or more (*p* < 0.01 for both thresholds; Table 3; Figure 3B). The absolute change in body weight from baseline to day 180 was −8.6 kg (95% CI: −9.4 kg to −7.8 kg) in homozygous minor allele carriers, compared with −4.9 kg (95% CI: −5.4 kg to −4.4 kg) in mixed allele carriers (treatment difference, −3.7 kg; 95% CI: −5.0 kg to −2.4 kg; *p* < 0.01).

### 3.2. Other Secondary End Points

Glucomannan, inulin, and psyllium were associated with greater reductions from baseline compared with placebo in BMI (−2.2 kg/m^2^ (95% CI: −2.3 to −2.1) in the experimental group vs. −0.8 kg/m^2^ (95% CI: −0.9 to −0.6) in the placebo group; treatment difference, −1.4 kg/m^2^ (95% CI: −1.7 to −1.2); *p* < 0.01), fat mass (−19.4% (95% CI: −21.3 to −17.5) in the experimental group vs. −6.4% (95% CI: −6.9 to −5.8) in the placebo group; treatment difference, −13.0% (95% CI: −14.4 to −11.7); *p* < 0.01), and visceral fat rating (−1.9 (95% CI: −2.1 to −1.7) in the experimental group vs. −0.6 (95% CI: −1.1 to −0.1) in the placebo group; treatment difference, −1.3 (95% CI: −1.6 to −1.0); *p* < 0.01; Table 2).

Moreover, there were no significant differences between the groups in changes to fat-free mass, blood pressure, fasting plasma glucose, hsCRP, and lipid profiles, including total cholesterol, LDL-C, HDL-C, and triglycerides, from baseline to day 180.

In the subgroup analysis, homozygous minor allele carriers exhibited a more significant reduction in BMI (−2.9 kg/m^2^ (95% CI: −3.4 to −2.4) in homozygous minor allele carriers vs. −1.7 kg/m^2^ (95% CI: −2.0 to −1.4) in mixed allele carriers; treatment difference, −1.2 kg/m^2^ (95% CI: −2.0 to −0.4); *p* < 0.01), fat mass (−24.9% (95% CI: −27.2 to −22.6) in homozygous minor allele carriers vs. −15.1% (95% CI: −16.8 to −13.5) in mixed allele carriers; treatment difference, −9.8% (95% CI: −10.6 to −9.1); *p* < 0.01), and visceral fat rating (−2.4 (95% CI: −2.6 to −2.2) in homozygous minor allele carriers vs. −1.5 (95% CI: −1.7 to −1.3) in mixed allele carriers; treatment difference, −0.9 (95% CI: −1.3 to −0.5); *p* < 0.01; Table 3).

### 3.3. Postprandial Appetite

After a standardized breakfast, VAS ratings for hunger and prospective food consumption were significantly reduced, whereas fullness and satiety ratings significantly increased with glucomannan, inulin, and psyllium compared to placebo (*p* < 0.01 for all; Table 4). The overall postprandial appetite suppression score, calculated after the standardized breakfast, was significantly higher in the experimental group (25.6; 95% CI: 21.4 to 29.8) than in the placebo group (8.4; 95% CI: 6.1 to 10.7), with a treatment difference of 17.2 (95% CI: 15.3 to 19.1; *p* < 0.01).

In the subgroup analysis, the differences in hunger, fullness, satiety, and prospective food consumption ratings between homozygous minor allele carriers and mixed allele carriers did not reach statistical significance.

### 3.4. Safety and Side-Effect Profile

In our study, 74.6% of participants in the active group reported at least one adverse event, mainly mild-to-moderate gastrointestinal symptoms (Table 5). Specifically, 28.2% experienced mild flatulence and 9.9% moderate; 19.7% had mild abdominal discomfort with 7.0% moderate; and 15.5% reported mild altered bowel habits, with another 5.6% moderate. Conversely, the placebo group had minimal reports, with 5.9% experiencing mild flatulence, 2.9% mild abdominal discomfort, and 2.9% mild altered bowel habits, with no moderate symptoms reported. These side effects were transient and required no discontinuation of the supplement. Additionally, subgroup analysis showed that the occurrence of these symptoms did not significantly differ between homozygous minor allele carriers and mixed allele carriers.

## 4. Discussion

In our study, we specifically investigated the interaction between dietary fiber supplementation and genetic polymorphisms related to weight management. The primary outcomes of this randomized, double-blind, parallel-group clinical trial are significant: (1) Participants carrying minor alleles in genes such as FTO, LEP, LEPR, and MC4R demonstrated notable weight loss and appetite modification when consuming a combination of dietary fibers—glucomannan, inulin, and psyllium. (2) Those identified as homozygous minor allele carriers exhibited a more pronounced response in terms of weight reduction and appetite change compared to heterozygous and non-carriers of these polymorphisms. (3) Furthermore, the trial highlighted a significant prevalence of side effects, with 74.6% of participants in the active group reporting at least one adverse event. The nature and frequency of these gastrointestinal side effects, primarily mild to moderate, underscore the importance of considering tolerability alongside efficacy in fiber supplementation interventions.

These findings align with results from other clinical studies. For instance, the study by Pražnikar et al. indicated that dietary fiber supplements, in conjunction with energy restriction, significantly reduced body weight, BMI, fat mass, visceral fat, and improved lipid profile and inflammation markers. Notably, a supplement containing glucomannan, inulin, psyllium, and apple fiber was most effective in reducing BMI, body weight, and CRP levels, suggesting that dietary fiber supplements could enhance weight loss and positively impact metabolic health in individuals with obesity and overweight [37].

Additionally, research among pregnant women highlighted the importance of certain genetic polymorphisms, including rs9939609 of the FTO gene, rs7799039 of the LEP gene, and rs1137101 of the LEPR gene. These polymorphisms were linked to mechanisms regulating dietary consumption, such as total energy intake, food preferences, and satiety responsiveness [42]. These findings emphasize the potential role of genetic factors in weight regulation and may explain the varied responses to dietary fiber observed in our study.

Our study’s observations regarding the interaction of dietary fiber supplementation with genetic polymorphisms in weight management align well with the broader body of research in this area. For instance, a study by Hosseini-Esfahani et al. revealed that dietary fiber could modify the association of FTO single-nucleotide polymorphisms (SNPs) and genetic risk scores with obesity. This effect was more pronounced in subjects consuming higher levels of dietary fiber and with a high genetic risk score, highlighting the potential of dietary fiber in mitigating genetic predispositions to obesity. The study also identified a significant gene–fiber interaction in relation to abdominal obesity, particularly between fiber and rs3751812, suggesting that individuals with a higher number of risk alleles could benefit more from high dietary fiber intake [43].

In terms of mechanisms, dietary fiber has been shown to induce greater satiety compared to simple sugars, potentially due to its physical properties, such as bulking and alteration of the viscosity of gastric contents. This effect may delay gastric emptying, blunt postprandial glucose and insulin responses, and influence the secretion of gut peptide hormones that regulate satiation. Moreover, fermentable fibers are consumed by intestinal bacteria, producing short-chain fatty acids that impact gene expression, including genes associated with obesity and metabolic health. This complex interaction of dietary components and genetic factors underscores the nuanced nature of dietary guidelines, especially for individuals with genetic predispositions to obesity [44,45].

Further emphasizing the role of dietary patterns, adherence to the Mediterranean dietary pattern, rich in fiber, has shown interactions with the FTO-rs9939609, linking a low adherence to the diet with higher type 2 diabetes risk in risk allele carriers. This indicates that genetic predispositions may not fully dictate health outcomes and can be modulated by dietary habits. The consumption of unhealthy food groups, typically low in dietary fiber, has also been studied in relation to obesity, demonstrating that subjects with a higher number of FTO risk alleles might be more susceptible to obesity when consuming foods like fried foods and sugary beverages [46,47,48].

Our study contributes to the understanding of how dietary fiber supplementation, particularly with glucomannan, inulin, and psyllium, impacts weight management among individuals with specific obesity-associated genetic polymorphisms. While our findings indicate a more pronounced response in homozygous minor allele carriers, it is important to note that mixed allele carriers also benefited from the intervention. This suggests that while genetic makeup may influence the magnitude of response, the intervention itself is broadly beneficial. Caution should be exercised in interpreting these results as indicative of the need for genotype-specific dietary interventions. Further research is required to explore whether different diet regimens might differentially benefit individuals based on their genotype, fully realizing the potential of nutrigenomics in obesity management.

An important consideration raised by our findings is the high prevalence of side effects associated with fiber supplementation. While 74.6% of participants in the active group reported gastrointestinal symptoms, this figure is not without precedent in dietary fiber research. Comparable studies have reported varying degrees of gastrointestinal responses to fiber intake, which are thought to be a function of fiber type, dosage, and individual digestive tolerance [49]. The side effects noted in our study were largely mild-to-moderate and transient, aligning with the existing literature that suggests such reactions are typically short-lived and diminish as the body adapts to increased fiber intake [50].

However, the significant occurrence of these symptoms in our study raises questions about the balance between the benefits of fiber supplements in weight management and their tolerability. This aspect of fiber supplementation is often underreported and warrants further investigation, especially considering the potential influence of genetic variations. While our subgroup analysis did not find a significant difference in side effects between homozygous minor allele carriers and mixed allele carriers, this is an avenue for future research. Such studies could explore the genetic basis for the variability in fiber tolerance and the potential for personalizing fiber supplementation strategies to mitigate adverse effects.

For future research, our findings open avenues for exploring the intersection of nutrigenomics and dietetics. Further studies could investigate other genetic markers and their interaction with different dietary components, potentially leading to a broader understanding of personalized nutrition. Such research might also delve into the long-term effects of tailored dietary interventions based on genetic profiles and evaluate the efficacy of these interventions in diverse populations.

One of the primary strengths of our research lies in its methodological rigor. The randomized, double-blind, parallel-group design minimizes the risk of bias and confounding factors, thereby enhancing the reliability of the results. The inclusion of specific genetic polymorphisms, such as FTO, LEP, LEPR, and MC4R, adds a level of precision in understanding the interaction between genetic factors and dietary fiber supplementation. Additionally, the substantial size of our study population and the duration of the intervention (180 days) provide robust data for assessing the long-term effects of dietary fiber on weight management in a genetically diverse group.

However, the study also has limitations that warrant consideration. The selection of heterozygous individuals as controls, while deliberate for our research focus, may not fully represent the impact compared to non-carriers of the polymorphisms, potentially affecting the generalizability of the findings to broader populations. This specific methodological approach has been acknowledged in the limitations of our study, alongside the challenges of capturing the full spectrum of genetic variations related to obesity, potential unmeasured confounding variables, reliance on self-reported dietary data, and the representativeness of our study population in terms of age, ethnicity, and socio-demographic factors. Future research should consider these aspects, aiming to include a more diverse control group, a broader range of genetic markers, expanded demographic representation, and objective measures of dietary intake and adherence.

## 5. Conclusions

This study underscores the influence of dietary fibers (glucomannan, inulin, and psyllium) on weight management, particularly noting the variable responses linked to genetic polymorphisms. While homozygous minor allele carriers showed a more marked response, all genotype groups benefited, suggesting the potential of fiber supplements beyond a genotype-specific approach. The high prevalence of mild-to-moderate gastrointestinal side effects, however, underscores the need for careful consideration of tolerability in dietary interventions. These findings advocate for integrating genetic insights with individual tolerability profiles in developing tailored nutritional strategies for weight management.

Despite the study’s contributions, its limitations in participant demographics and duration warrant further, more diverse, and prolonged research. Future studies should also examine the long-term effects and mechanisms of dietary fibers across various genetic backgrounds, aiming to optimize the balance between efficacy and tolerability in personalized nutrition. This research enriches the field of nutritional genomics, paving the way for more nuanced and effective dietary recommendations in the realm of precision medicine.

## Figures and Tables

**Table 1:** Demographic and clinical characteristics of the participants at baseline.

	Glucomannan, Inulin, Psyllium (*n* = 75)	Placebo (*n* = 37)	*p*-Value	Homozygous Minor Allele Carriers (*n* = 33)	Mixed Allele Carriers (*n* = 42)	*p*-Value
Age, y	46 ± 10	46 ± 12	*p* = 0.81	45 ± 9	46 ± 11	*p* = 0.78
Women, %	71.8	72.0	*p* = 0.89	72.7	71.4	*p* = 0.82
Body weight, kg	89.3 ± 10.4	90.6 ± 10.8	*p* = 0.73	91.4 ± 11.1	87.8 ± 9.6	*p* = 0.13
Body mass index, kg/m^2^	30.7 ± 3.4	31.3 ± 3.5	*p* = 0.67	31.1 ± 3.8	30.5 ± 2.9	*p* = 0.11
Fat mass, %	37.5 ± 4.6	38.1 ± 4.2	*p* = 0.46	37.8 ± 3.8	37.1 ± 5.1	*p* = 0.58
Fat-free mass, kg	57.5 ± 10.7	57.1 ± 11.8	*p* = 0.72	56.1 ± 11.2	58.3 ± 10.1	*p* = 0.32
Visceral fat rating	9.7 ± 3.5	9.5 ± 3.6	*p* = 0.68	9.8 ± 3.5	9.6 ± 3.4	*p* = 0.75
Systolic blood pressure, mm Hg	128 ± 9	129 ± 9	*p* = 0.88	129 ± 8	128 ± 11	*p* = 0.61
Diastolic blood pressure, mm Hg	81 ± 7	82 ± 4	*p* = 0.71	82 ± 6	81 ± 6	*p* = 0.74
Total cholesterol, mmol/L	5.12 ± 0.73	5.1 ± 0.7	*p* = 0.90	5.32 ± 0.62	4.98 ± 0.78	*p* = 0.14
LDL-C, mmol/L	3.95 ± 0.71	4.18 ± 0.59	*p* = 0.86	4.14 ± 0.48	3.82 ± 0.91	*p* = 0.13
HDL-C, mmol/L	1.54 ± 0.46	1.59 ± 0.52	*p* = 0.76	1.44 ± 0.43	1.61 ± 0.48	*p* = 0.23
Triglycerides, mmol/L	1.28 ± 0.57	1.37 ± 0.64	*p* = 0.78	1.38 ± 0.51	1.22 ± 0.63	*p* = 0.16
Fasting plasma glucose, mmol/L	5.55 ± 0.50	5.68 ± 0.58	*p* = 0.64	5.39 ± 0.41	5.68 ± 0.59	*p* = 0.12
hsCRP, mg/L	2.00 ± 1.28	2.32 ± 1.3	*p* = 0.78	2.11 ± 1.35	1.92 ± 1.21	*p* = 0.33

**Table 2:** Primary and secondary endpoints at 180-day follow-up in main groups.

	Glucomannan, Inulin, Psyllium (*n* = 71)	Placebo (*n* = 34)	Difference (95% CI)	Odds Ratio	*p*-Value
Body weight change from baseline to day 180, %	−7.3 (−9.0 to −5.6)	−2.4 (−3.5 to −1.3)	−4.9 (−6.9 to −2.9)		*p* < 0.01
Participants with body weight reduction ≥ 5% at day 180, %	59.2	26.5		4.0 (1.6 to 9.9)	*p* < 0.01
Participants with body weight reduction ≥ 10% at day 180, %	21.1	8.8		2.8 (1.4 to 5.4)	*p* < 0.01
Body weight (change from baseline to day 180), kg	−6.5 (−7.2 to −5.9)	−2.2 (−2.4 to −2.0)	−4.3 (−5.2 to −3.5)		*p* < 0.01
Body mass index (change from baseline to day 180), kg/m^2^	−2.2 (−2.3 to −2.1)	−0.8 (−0.9 to −0.6)	−1.4 (−1.7 to −1.2)		*p* < 0.01
Fat mass, (change from baseline to day 180), %	−19.4 (−21.3 to −17.5)	−6.4 (−6.9 to −5.8)	−13.0 (−14.4 to −11.7)		*p* < 0.01
Fat-free mass (change from baseline to day 180), kg	−1.3 (−4.9 to 2.3)	−0.8 (−3.0 to 1.4)	−0.5 (−6.3 to 5.3)		*p* = 0.11
Visceral fat rating (change from baseline to day 180)	−1.9 (−2.1 to −1.7)	−0.6 (−1.1 to −0.1)	−1.3 (−1.6 to −1.0)		*p* < 0.01
Systolic blood pressure, mm Hg	−4.8 (−10.3 to −1.3)	−9.1 (−13.7 to −4.5)	4.2 (−1.7 to 10.1)		*p* = 0.13
Diastolic blood pressure, mm Hg	−3.9 (−7.5 to −0.4)	−7.3 (−9.7 to −4.9)	3.1 (−1.2 to 7.4)		*p* = 0.15
Total cholesterol (ratio of day-180 value to baseline)	0.93 (0.75 to 1.11)	0.92 (0.74 to 1.10)	1.01 (0.82 to 1.21)		*p* = 0.46
LDL-C (ratio of day-180 value to baseline)	0.92 (0.74 to 1.08)	0.91 (0.73 to 1.09)	1.01 (0.81 to 1.21)		*p* = 0.51
HDL-C (ratio of day-180 value to baseline)	0.91 (0.73 to 1.08)	0.97 (0.78 to 1.16)	0.94 (0.75 to 1.15)		*p* = 0.05
Triglycerides (ratio of day-180 value to baseline)	0.93 (0.77 to 1.13)	0.82 (0.66 to 0.98)	1.13 (0.93 to 1.38)		*p* = 0.10
hsCRP (ratio of day-180 value to baseline)	0.91 (0.75 to 1.07)	1.16 (0.99 to 1.33)	1.03 (0.85 to 1.21)		*p* = 0.08
Fasting plasma glucose, mmol/L	−0.38 (−1.56 to 0.80)	−0.48 (−0.94 to −0.02)	0.10 (−1.15 to 1.35)		*p* = 0.26

**Table 3:** Secondary outcome measures at 180-day follow-up for homozygous and mixed minor allele carriers receiving glucomannan, inulin, and psyllium.

	Homozygous Minor Allele Carriers (*n* = 31)	Mixed Allele Carriers (*n* = 40)	Difference between Homozygous and Mixed Allele (95% CI)	Odds Ratio	*p*-Value
Body weight change from baseline to day 180, %	−9.4 (−10.6 to −8.2)	−5.6 (−6.7 to −4.4)	−3.2 (−4.9 to −1.6)		*p* < 0.01
Participants with body weight reduction ≥ 5% at day 180, %	77.4	45.0		4.1 (2.7 to 6.0)	*p* < 0.01
Participants with body weight reduction ≥ 10% at day 180, %	29.0	15.0		2.3 (1.5 to 3.4)	*p* < 0.01
Body weight (change from baseline to day 180), kg	−8.6 (−9.4 to −7.8)	−4.9 (−5.4 to −4.4)	−3.7 (−5.0 to −2.4)		*p* < 0.01
Body mass index (change from baseline to day 180), kg/m^2^	−2.9 (−3.4 to −2.4)	−1.7 (−2.0 to −1.4)	−1.2 (−2.0 to −0.4)		*p* < 0.01
Fat mass, (change from baseline to day 180), %	−24.9 (−27.2 to −22.6)	−15.1 (−16.6 to −13.5)	−9.8% (−10.6% to −9.1%)		*p* < 0.01
Fat-free mass (change from baseline to day 180), kg	−1.7 (−5.7 to 2.3)	−1.0 (−4.3 to 2.3)	−0.7 (−8.0 to 6.6)		*p* = 0.09
Visceral fat rating (change from baseline to day 180)	−2.4 (−2.6 to −2.2)	−1.5 (−1.7 to −1.3)	−0.9 (−1.3 to −0.5)		*p* < 0.01
Systolic blood pressure (change from baseline to day 180), mm Hg	−7.1 (−10.4 to −3.8)	−2.9 (−6.4 to 0.6)	−4.2 (−8.0 to −0.4)		*p* = 0.07
Diastolic blood pressure (change from baseline to day 180), mm Hg	−5.1 (−7.6 to −2.6)	−3.2 (−5.4 to −1.0)	−1.9 (−5.2 to 1.4)		*p* = 0.28
Total cholesterol (ratio of day-180 value to baseline)	0.94 (0.76 to 1.12)	0.91 (0.73 to 1.09)	1.0 (0.79 to 1.21)		*p* = 0.32
LDL-C (ratio of day-180 value to baseline)	0.93 (0.75 to 1.11)	0.90 (0.74 to 1.06)	1.02 (0.81 to 1.23)		*p* = 0.25
HDL-C (ratio of day-180 value to baseline)	0.92 (0.74 to 1.10)	0.89 (0.72 to 1.06)	0.03 (0.01 to 0.05)		*p* = 0.60
Triglycerides (ratio of day-180 value to baseline)	0.85 (0.69 to 1.01)	1.04 (0.84 to 1.24)	−0.19 (−0.40 to 0.02)		*p* = 0.10
hsCRP (ratio of day-180 value to baseline)	0.79 (0.64 to 0.94)	1.04 (0.84 to 1.24)	−0.25 (−0.45 to −0.05)		*p* = 0.06
Fasting plasma glucose (change from baseline to day 180), mmol/L	−0.49 (−0.95 to −0.03)	−0.27 (−1.35 to 0.81)	−0.22 (−1.40 to 0.96)		*p* = 0.12

**Table 4:** Postprandial appetite ratings after standardized breakfast at 180-day follow-up, showing absolute changes from pre-breakfast (fasting) VAS scores.

	Glucomannan, Inulin, Psyllium (*n* = 71)	Placebo (*n* = 34)	Difference (95% CI)	*p*-Value
Hunger	−12.3 (−15.2 to −9.4)	−3.1 (−5.0 to −1.2)	−9.2 (−11.4 to −7.0)	*p* < 0.01
Fullness	10.4 (7.5 to 13.3)	2.1 (0 to 4.2)	8.3 (6.1 to 10.5)	*p* < 0.01
Satiety	11.6 (8.4 to 14.8)	4.2 (2.1 to 6.3)	7.4 (5.2 to 9.6)	*p* < 0.01
Prospective	−10.7 (−13.5 to −7.9)	−2.3 (−4.0 to 0)	−8.4 (−10.6 to −6.2)	*p* < 0.01
Overall appetite suppression score	25.6 (21.4 to 29.8)	8.4 (6.1 to 10.7)	17.2 (15.3 to 19.1)	*p* < 0.01

**Table 5:** Prevalence and severity of reported adverse events in active and placebo groups.

	Glucomannan, Inulin, Psyllium (*n* = 71)		Placebo (*n* = 34)		*p*-Value
	% Mild	% Moderate	% Mild	% Moderate	
Flatulence, % (*n*)	28.2% (20)	9.9% (9)	5.9% (2)	0% (0)	<0.01
Abdominal Discomfort, % (*n*)	19.7% (14)	7.0% (5)	2.9% (1)	0% (0)	<0.01
Altered Bowel Habits, % (*n*)	15.5% (11)	5.6% (4)	2.9% (1)	0% (0)	<0.01

## Data Availability

The data presented in this study are available on request from the corresponding author. The data are not publicly available as the participants did not consent to their data being shared publicly.

## References

[1] NCD Risk Factor Collaboration (2017). Worldwide trends in body-mass index, underweight, overweight, and obesity from 1975 to 2016: A pooled analysis of 2416 population-based measurement studies in 128.9 million children, adolescents, and adults. Lancet.

[2] Pagotto U., Vanuzzo D., Vicennati V., Pasquali R. (2008). Pharmacological therapy of obesity. G. Ital. Cardiol..

[3] Fried M., Hainer V., Basdevant A., Buchwald H., Deitel M., Finer N., Greve J.W.M., Horber F., Mathus-Vliegen E., Scopinaro N. (2008). Interdisciplinary European guidelines on surgery of severe obesity. Obes. Facts.

[4] Artham S.M., Lavie C.J., Milani R.V., Ventura H.O. (2008). The obesity paradox: Impact of obesity on the prevalence and prognosis of cardiovascular diseases. Postgrad. Med..

[5] Abdelaal M., le Roux C.W., Docherty N.G. (2017). Morbidity and mortality associated with obesity. Ann. Transl. Med..

[6] Kushner R.F., Ryan D.H. (2014). Assessment and lifestyle management of patients with obesity: Clinical recommendations from systematic reviews. JAMA.

[7] Corella D., Qi L., Sorlí J.V., Godoy D., Portolés O., Coltell O., Greenberg A.S., Ordovas J.M. (2005). Obese subjects carrying the 11482G > A polymorphism at the perilipin locus are resistant to weight loss after dietary energy restriction. J. Clin. Endocrinol. Metab..

[8] Phares D.A., Halverstadt A.A., Shuldiner A.R., Ferrell R.E., Douglass L.W., Ryan A.S., Goldberg A.P., Hagberg J.M. (2004). Association between body fat response to exercise training and multilocus ADR genotypes. Obes. Res..

[9] MacLean P.S., Wing R.R., Davidson T., Epstein L., Goodpaster B., Hall K.D., Levin B.E., Perri M.G., Rolls B.J., Rosenbaum M. (2015). NIH working group report: Innovative research to improve maintenance of weight loss. Obesity.

[10] Apovian C.M., Aronne L.J., Bessesen D.H., McDonnell M.E., Murad M.H., Pagotto U., Ryan D.H., Still C.D., Endocrine S. (2015). Pharmacological management of obesity: An endocrine Society clinical practice guideline. J. Clin. Endocrinol. Metab..

[11] Patel D. (2015). Pharmacotherapy for the management of obesity. Metab. Clin. Exp..

[12] Jovanovski E., Mazhar N., Komishon A., Khayyat R., Li D., Blanco Mejia S., Khan T., Jenkins A.L., Smircic-Duvnjak L., Sievenpiper J.L. (2020). Can dietary viscous fiber affect body weight independently of an energy-restrictive diet? A systematic review and meta-analysis of randomized controlled trials. Am. J. Clin. Nutr..

[13] Malnick S.D.H., Knobler H. (2006). The medical complications of obesity. QJM Mon. J. Assoc. Physicians.

[14] Reynolds A., Mann J., Cummings J., Winter N., Mete E., Te Morenga L. (2019). Carbohydrate quality and human health: A series of systematic reviews and meta-analyses. Lancet.

[15] Slavin J.L. (2005). Dietary fiber and body weight. Nutrition.

[16] Thompson S.V., Hannon B.A., An R., Holscher H.D. (2017). Effects of isolated soluble fiber supplementation on body weight, glycemia, and insulinemia in adults with overweight and obesity: A systematic review and meta-analysis of randomized controlled trials. Am. J. Clin. Nutr..

[17] Fuglsang-Nielsen R., Rakvaag E., Langdahl B., Knudsen K.E.B., Hartmann B., Holst J.J., Hermansen K., Gregersen S. (2021). Effects of whey protein and dietary fiber intake on insulin sensitivity, body composition, energy expenditure, blood pressure, and appetite in subjects with abdominal obesity. Eur. J. Clin. Nutr..

[18] Lyon M.R., Reichert R.G. (2010). The effect of a novel viscous polysaccharide along with lifestyle changes on short-term weight loss and associated risk factors in overweight and obese adults: An observational retrospective clinical program analysis. Altern. Med. Rev. A J. Clin. Ther..

[19] Cairella M., Marchini G. (1995). Evaluation of the action of glucomannan on metabolic parameters and on the sensation of satiation in overweight and obese patients. Clin. Ter..

[20] Hess A.L., Benítez-Páez A., Blædel T., Larsen L.H., Iglesias J.R., Madera C., Sanz Y., Larsen T.M., MyNewGut C. (2020). The effect of inulin and resistant maltodextrin on weight loss during energy restriction: A randomised, placebo-controlled, double-blinded intervention. Eur. J. Nutr..

[21] Qin Y.-Q., Wang L.-Y., Yang X.-Y., Xu Y.-J., Fan G., Fan Y.-G., Ren J.-N., An Q., Li X. (2023). Inulin: Properties and health benefits. Food Funct..

[22] Brighenti F. (2007). Dietary fructans and serum triacylglycerols: A meta-analysis of randomized controlled trials. J. Nutr..

[23] McRorie J.W., McKeown N.M. (2017). Understanding the Physics of Functional Fibers in the Gastrointestinal Tract: An Evidence-Based Approach to Resolving Enduring Misconceptions about Insoluble and Soluble Fiber. J. Acad. Nutr. Diet..

[24] Blackwood A.D., Salter J., Dettmar P.W., Chaplin M.F. (2000). Dietary fibre, physicochemical properties and their relationship to health. J. R. Soc. Promot. Health.

[25] Yao M., Roberts S.B. (2001). Dietary energy density and weight regulation. Nutr. Rev..

[26] Holt S., Brand J., Soveny C., Hansky J. (1992). Relationship of satiety to postprandial glycaemic, insulin and cholecystokinin responses. Appetite.

[27] Burton-Freeman B., Davis P.A., Schneeman B.O. (2002). Plasma cholecystokinin is associated with subjective measures of satiety in women. Am. J. Clin. Nutr..

[28] Anderson J.W., Randles K.M., Kendall C.W.C., Jenkins D.J.A. (2004). Carbohydrate and fiber recommendations for individuals with diabetes: A quantitative assessment and meta-analysis of the evidence. J. Am. Coll. Nutr..

[29] Goodarzi M.O. (2018). Genetics of Obesity: What Genetic Association Studies Have Taught Us about the Biology of Obesity and Its Complications. Lancet Diabetes Endocrinol..

[30] Leonska-Duniec A., Jastrzebski Z., Jazdzewska A., Krzysztof F., Cieszczyk P. (2018). Leptin and Leptin Receptor Genes Are Associated With Obesity-Related Traits Changes in Response to Aerobic Training Program. J. Strength Cond. Res..

[31] Paolin B., Maltese P.E., Del Ciondolo I., Tavian D., Missaglia S., Ciuoli C., Zuntini M., Cecchin S., Bertelli M., Pompucci G. (2016). Prevalence of mutations in LEP, LEPR, and MC4R genes in individuals with severe obesity. Genet. Mol. Res..

[32] Loos R.J.F., Yeo G.S.H. (2014). The Bigger Picture of FTO: The First GWAS-Identified Obesity Gene. Nat. Rev. Endocrinol..

[33] Fairbrother U., Kidd E., Malagamuwa T., Walley A. (2018). Genetics of Severe Obesity. Curr. Diab. Rep..

[34] Hertzel A.V., Bernlohr D.A. (2000). The Mammalian Fatty Acid-Binding Protein Multigene Family: Molecular and Genetic Insights into Function. Trends Endocrinol. Metab..

[35] Evans D.S., Calton M.A., Kim M.J., Kwok P.-Y., Miljkovic I., Harris T., Koster A., Liu Y., Tranah G.J., Ahituv N. (2014). Genetic Association Study of Adiposity and Melanocortin-4 Receptor (MC4R) Common Variants: Replication and Functional Characterization of Non-Coding Regions. PLoS ONE.

[36] Leonska-Duniec A., Jastrzebski Z., Zarebska A., Smółka W., Cieszczyk P. (2017). Impact of the Polymorphism Near MC4R (Rs17782313) on Obesity-and Metabolic-Related Traits inWomen Participating in an Aerobic Training Program. J. Hum. Kinet..

[37] Pražnikar Z.J., Mohorko N., Gmajner D., Kenig S., Petelin A. (2023). Effects of Four Different Dietary Fibre Supplements on Weight Loss and Lipid and Glucose Serum Profiles during Energy Restriction in Patients with Traits of Metabolic Syndrome: A Comparative, Randomized, Placebo-Controlled Study. Foods.

[38] Maculewicz E., Leońska-Duniec A., Mastalerz A., Szarska E., Garbacz A., Lepionka T., Łakomy R., Anyżewska A., Bertrandt J. (2022). The Influence of FTO, FABP2, LEP, LEPR, and MC4R Genes on Obesity Parameters in Physically Active Caucasian Men. Int. J. Environ. Res. Public. Health..

[39] Sood N., Baker W., Coleman C. (2008). Effect of glucomannan on plasma lipid and glucose concentrations, body weight, and blood pressure: Systematic review and meta-analysis. Am. J. Clin. Nutr..

[40] Salazar N., Dewulf E., Neyrinck A., Bindels L., Cani P., Mahillon J., de Vos W., Thissen J., Gueimonde M., de Los Reyes-Gavilán C. (2015). Inulin-type fructans modulate intestinal Bifidobacterium species populations and decrease fecal short-chain fatty acids in obese women. Clin. Nutr..

[41] Anderson J., Allgood L., Lawrence A., Altringer L., Jerdack G., Hengehold D., Morel J. (2000). Cholesterol-lowering effects of psyllium intake adjunctive to diet therapy in men and women with hypercholesterolemia: Meta-analysis of 8 controlled trials. Am. J. Clin. Nutr..

[42] Martins M.C., Trujillo J., Freitas-Vilela A.A., Farias D.R., Rosado E.L., Struchiner C.J., Kac G. (2018). Associations between obesity candidate gene polymorphisms (fat mass and obesity-associated (FTO), melanocortin-4 receptor (MC4R), leptin (LEP) and leptin receptor (LEPR)) and dietary intake in pregnant women. Br. J. Nutr..

[43] Hosseini-Esfahani F., Koochakpoor G., Daneshpour M.S., Mirmiran P., Sedaghati-Khayat B., Azizi F. (2017). The interaction of fat mass and obesity associated gene polymorphisms and dietary fiber intake in relation to obesity phenotypes. Sci. Rep..

[44] Warrilow A., Mellor D., McKune A., Pumpa K. (2019). Dietary fat, fibre, satiation, and satiety-a systematic review of acute studies. Eur. J. Clin. Nutr..

[45] Vella A., Camilleri M. (2017). The Gastrointestinal Tract as an Integrator of Mechanical and Hormonal Response to Nutrient Ingestion. Diabetes.

[46] Razquin C., Martinez J.A., Martinez-Gonzalez M.A., Bes-Rastrollo M., Fernández-Crehuet J., Marti A. (2010). A 3-year intervention with a Mediterranean diet modified the association between the rs9939609 gene variant in FTO and body weight changes. Int. J. Obes..

[47] Ortega-Azorín C., Sorlí J.V., Asensio E.M., Coltell O., Martínez-González M.Á., Salas-Salvadó J., Covas M.I., Arós F., Lapetra J., Serra-Majem L. (2012). Associations of the FTO rs9939609 and the MC4R rs17782313 polymorphisms with type 2 diabetes are modulated by diet, being higher when adherence to the Mediterranean diet pattern is low. Cardiovasc. Diabetol..

[48] Di Renzo L., Cioccoloni G., Falco S., Abenavoli L., Moia A., Salimei P.S., De Lorenzo A. (2018). Influence of FTO rs9939609 and Mediterranean diet on body composition and weight loss: A randomized clinical trial. J. Transl. Med..

[49] Grundy M.M.-L., Edwards C.H., Mackie A.R., Gidley M.J., Butterworth P.J., Ellis P.R. (2016). Re-evaluation of the mechanisms of dietary fibre and implications for macronutrient bioaccessibility, digestion and post-prandial metabolism. Br. J. Nutr..

[50] Lovegrove A., Edwards C.H., De Noni I., Patel H., El S.N., Grassby T., Zielke C., Ulmius M., Nilsson L., Butterworth P.J. (2017). Role of polysaccharides in food, digestion, and health. Crit. Rev. Food Sci. Nutr..

